# Respiratory gas kinetics in patients with congestive heart failure during recovery from peak exercise

**DOI:** 10.1016/j.clinsp.2023.100225

**Published:** 2023-06-23

**Authors:** Alessandro Patti, Yair Blumberg, Kristofer Hedman, Daniel Neunhäuserer, Francois Haddad, Matthew Wheeler, Euan Ashley, Kegan J. Moneghetti, Jonathan Myers, Jeffrey W. Christle

**Affiliations:** a Division of Cardiovascular Medicine, Department of Medicine, Stanford, California, USA; b Division of Sports and Exercise Medicine, Department of Medicine, University of Padova, Padova, Italy; c Azrieli Faculty of Medicine, Bar-Ilan University, Safed, Israel; d Stanford Sports Cardiology, Stanford University, Stanford, California, USA; e Department of Clinical Physiology, and Department of Health, Medicine and Caring Sciences, Linköping University, Linköping, Sweden; f Baker Department of Cardiometabolic Health, University of Melbourne, Australia; g National Centre for Sports Cardiology, St Vincent's Hospital, Melbourne, Australia; h Division of Cardiology, Veterans Affairs Palo Alto Health Care System, Palo Alto, California, USA

**Keywords:** Respiratory exchange ratio (RER), Cardiopulmonary exercise testing, Oxygen uptake, Carbon dioxide production, Overshoot

## Abstract

**Background:**

Cardiopulmonary Exercise Testing (CPX) is essential for the assessment of exercise capacity for patients with Chronic Heart Failure (CHF). Respiratory gas and hemodynamic parameters such as Ventilatory Efficiency (VE/VCO_2_ slope), peak oxygen uptake (peak VO_2_), and heart rate recovery are established diagnostic and prognostic markers for clinical populations. Previous studies have suggested the clinical value of metrics related to respiratory gas collected during recovery from peak exercise, particularly recovery time to 50% (T1/2) of peak VO_2_. The current study explores these metrics in detail during recovery from peak exercise in CHF.

**Methods:**

Patients with CHF who were referred for CPX and healthy individuals without formal diagnoses were assessed for inclusion. All subjects performed CPX on cycle ergometers to volitional exhaustion and were monitored for at least five minutes of recovery. CPX data were analyzed for overshoot of respiratory exchange ratio (RER=VCO_2_/VO_2_), ventilatory equivalent for oxygen (VE/VO_2_), end-tidal partial pressure of oxygen (PETO_2_), and T1/2 of peak VO_2_ and VCO_2_.

**Results:**

Thirty-two patients with CHF and 30 controls were included. Peak VO_2_ differed significantly between patients and controls (13.5 ± 3.8 vs. 32.5 ± 9.8 mL/Kg*min^−1^, p < 0.001). Mean Left Ventricular Ejection Fraction (LVEF) was 35.9 ± 9.8% for patients with CHF compared to 61.1 ± 8.2% in the control group. The T1/2 of VO_2_, VCO_2_ and VE was significantly higher in patients (111.3 ± 51.0, 132.0 ± 38.8 and 155.6 ± 45.5s) than in controls (58.08 ± 13.2, 74.3 ± 21.1, 96.7 ± 36.8s; p < 0.001) while the overshoot of PETO_2_, VE/VO_2_ and RER was significantly lower in patients (7.2 ± 3.3, 41.9 ± 29.1 and 25.0 ± 13.6%) than in controls (10.1 ± 4.6, 62.1 ± 17.7 and 38.7 ± 15.1%; all p < 0.01). Most of the recovery metrics were significantly correlated with peak VO_2_ in CHF patients, but not with LVEF.

**Conclusions:**

Patients with CHF have a significantly blunted recovery from peak exercise. This is reflected in delays of VO_2_, VCO_2_, VE, PETO_2_, RER and VE/VO_2_, reflecting a greater energy required to return to baseline. Abnormal respiratory gas kinetics in CHF was negatively correlated with peak VO_2_ but not baseline LVEF.

## Highlights

• Analysis of recovery from cardiopulmonary exercise testing reveals pathophysiology.
• Recovery of respiratory gases from peak exercise is hindered in heart failure.
• Recovery from peak exercise in heart failure is negatively correlated with peak VO_2_ but not baseline LVEF.

## Background

Cardiopulmonary Exercise Testing (CPX) provides substantial information on cardiopulmonary function and cardiorespiratory fitness through the evaluation of respiratory gases, ventilation, electrocardiographic and blood pressure adaptations, and symptoms during exercise. It is recommended in the diagnostic and prognostic work-up for several cardiac and pulmonary conditions as well as for clinical exercise prescription.^1^, ^2^, ^3^

The most established clinical CPX metrics collected during exercise are peak oxygen uptake (peak VO_2_), ventilatory efficiency (VE/VCO_2_ slope), and the pattern of end-tidal partial pressure of CO_2_ (PETCO_2_), which all have demonstrated strong independent diagnostic and prognostic value in patients with CHF.^1^ Combining several of these CPX metrics, different risk stratification scores have been developed which showed good validity in clinical applications.^4^, ^5^, ^6^, ^7^, ^8^ Recovery from exercise has been less thoroughly studied, with heart rate recovery being the only well-established metric from recovery for the risk stratification of patients referred for exercise testing. Assessment of respiratory gas kinetics during recovery may provide additional information and improve current methods for assessing prognosis in CHF.

It has been observed in small cohorts that the time of recovery of VO_2_ after exercise may be delayed in patients with CHF.^9^^,^^10^ Other authors have also described that prolonged recovery of cardiac output coincides with the prolonged recovery of VO_2_.^11^^,^^12^

Additional studies have observed that a marked delay in respiratory gas exchange kinetics post-exercise is correlated with worse clinical outcomes.^13^, ^14^, ^15^, ^16^, ^17^ More recently, other early recovery indices have been studied, such as the respiratory exchange ratio (RER = VCO_2_/VO_2_), ventilatory equivalent for oxygen (VE/VO_2_), and the end-tidal partial pressure of oxygen (PETO_2_). The increase (“overshoot”) of these parameters during recovery has been observed to be reduced in patients with CHF compared to healthy subjects, and furthermore to be correlated with other common markers of CRF.^18^^,^^19^

The aim of the present study was to comprehensively investigate the behavior of respiratory gas exchange kinetics during recovery from peak exercise in a population of patients with documented CHF compared to healthy controls.

## Methods

This study included patients with CHF and subjects with no history of the disease. All adult individuals who performed CPX in the Division of Cardiovascular Medicine of Stanford University Hospital between 2018‒2019 were eligible for the study. Patients were included in the experimental group if they had documented CHF, were between 18 and 75 years old, had no documented pulmonary or other relevant comorbidities, and performed successful CPX with at least five minutes of recovery. Healthy controls were recruited prospectively from the local community and were included if they did not have documented or reasonable suspicion of cardiovascular or pulmonary disease.

Exclusion criteria for exercise testing were: (i) Acute myocardial infarction; (ii) Unstable angina; (iii) Uncontrolled arrhythmia with hemodynamic compromise; (iv) Active/acute endocarditis, myocarditis, pericarditis; (v) Symptomatic severe aortic stenosis; (vi) Decompensated heart failure; (vii) Acute pulmonary injury or deep vein thrombosis; (viii) Acute aortic dissection; (ix) Any disability/comorbidity which precluded safe exercise testing; and (x) Inability to understand instructions and/or give informed consent.

Maximal exercise testing was performed on a cycle-ergometer (Ergoselect 100, Ergoline GmbH, Mitz, Germany) with respiratory gas collection using a metabolic cart (Quark CPET, CosMed SrI, Rome, Italy). The metabolic gas analyzers and turbines were calibrated before every exercise test. Respiratory gases were collected on a breath-by-breath basis and analysis was performed after applying a rolling average filter every 10 seconds averaged over the previous 30 seconds (Omnia 2.0, CosMed SrI, Rome, Italy). Heart rate was collected continuously with an integrated 12-lead ECG. Blood pressure was measured at rest, every two minutes during exercise, at peak intensity, and every two minutes during recovery. Peak VO_2_ was defined as the highest value attained in a 20‒30 second interval during the last phase of exercise per recommendations.^20^ The first ventilatory threshold (AT) was identified using the V-Slope method and visually confirmed.^21^ The Respiratory Compensation Point (RCP) was evaluated considering the simultaneous behavior of ventilatory equivalents and PETO_2_/PETCO_2_. The VE/VCO_2_ slope was calculated as the coefficient of linear regression obtained by plotting the VE and VCO_2_ data for the entire exercise phase. Hemodynamic and ventilatory parameters from CPX were visually inspected by two of the investigators, with disagreements being resolved by a third (AP, KM, JWC).

Recovery was defined as the period beginning when the workload was removed until participants returned to within 10% of resting HR and VO_2_. The recovery of respiratory gases was analyzed as a percentage change from the peak and as a time to 50% (T1/2) of peak VO_2_ after exercise over a duration of five minutes. The percentage change from peak values of the metrics displaying an overshoot during recovery (RER, VE/VO_2_ and PETO_2_) was calculated as the magnitude of the overshoot (i.e., the maximum percentage increase from peak).^19^ The half-time of recovery for respiratory metrics (VO_2_, VCO_2_ and VE) was defined as the time required to reach 50% of peak.

### Statistical analysis

The normality of the data was assessed using a visual inspection of frequencies and the Shapiro-Wilk test. Data are expressed as mean ± SD. Categorical variables are expressed as numbers and percentages. The difference between groups was assessed with a *t*-test for normally distributed variables and a Mann-Whitney *U* test for non-normally distributed variables. Correlations were evaluated with Pearson's correlation index if normally distributed and Spearman's correlation index if non-normally distributed. Statistical analysis was performed using IBM SPSS Statistics software version 25.

## Results

Table 1 shows the main characteristics of the two study groups. 32 patients with CHF and 30 controls were included in the study. Patients with CHF had predominantly a reduced EF (n = 29, 91%). Baseline demographics were not significantly different between the groups, with the exception of BMI which was higher in CHF patients (27.9 ± 5.0 vs. 24.5 ± 2.9 kg/m^2^, p < 0.01). Patients with CHF exercised for a shorter duration and to a lower peak load compared to controls (431 ± 146 vs. 615 ± 166s and 77±29 vs. 223 ± 73 W respectively; p < 0.01 for both comparisons). Peak VO_2,_ VE and the VE/VCO_2_ slope were significantly different between groups. Four patients and one control had significantly less than five minutes of recovery upon analyses (110s, 150s, 180s, 190s and 110s, respectively). The mean time of recorded recovery was 249 ± 40s for patients and 264 ± 29s for healthy subjects (p < 0.01).

**Table 1:** Characteristics of the study participants.

	CHF	Controls	p-value
**Sex (F)**	11 (34.4%)	14 (46.7%)	0.32
**Age (yrs)**	46.8 ± 13.0	43.1 ± 12.2	0.24
**BMI (Kg/m^2^)**	27.9 ± 5.0	24.5 ± 2.9	0.002
**Peak VO_2_ (mL/min)**	1134.9 ± 419.4	2407.5 ± 787.2	<0.001
Peak VO_2_ (mL/Kg*min^−1^)	13.5 ± 3.8	32.5 ± 9.8	<0.001
**Peak RER**	1.04 ± 0.09	1.16 ± 0.08	<0.001
**LVEF (%)**	35.9 ± 9.8	61.1 ± 8.2	<0.001
**Weber's Class**			
A	1 (3%)		
B	10 (31%)		
C	13 (41%)		
D	8 (25%)		
**Peak VCO_2_ (mL/min)**	1196.7 ± 492.7	2783.5 ± 887.0	<0.001
**Peak VE (L/min)**	42.5 ± 16.1	89.1 ± 33.1	<0.001
**VE/VCO_2_ slope**	37.1 ± 10.7	29.7 ± 4.0	<0.001
**VO_2_/HR**	9.7 ± 3.1	14.7 ± 4.5	<0.001

CHF, Congestive Heart Failure; BMI, Body Mass Index; VO_2_, Oxygen uptake; RER, Respiratory Exchange Ratio; LVEF, Left Ventricular Ejection Fraction; VCO_2_, Carbon Dioxide Production; VE, Tidal Ventilation; Weber Class: A, Little or no impairment; B, Mild to moderate impairment; C, Moderate to severe impairment; D, Severe.

**Figure 1:**
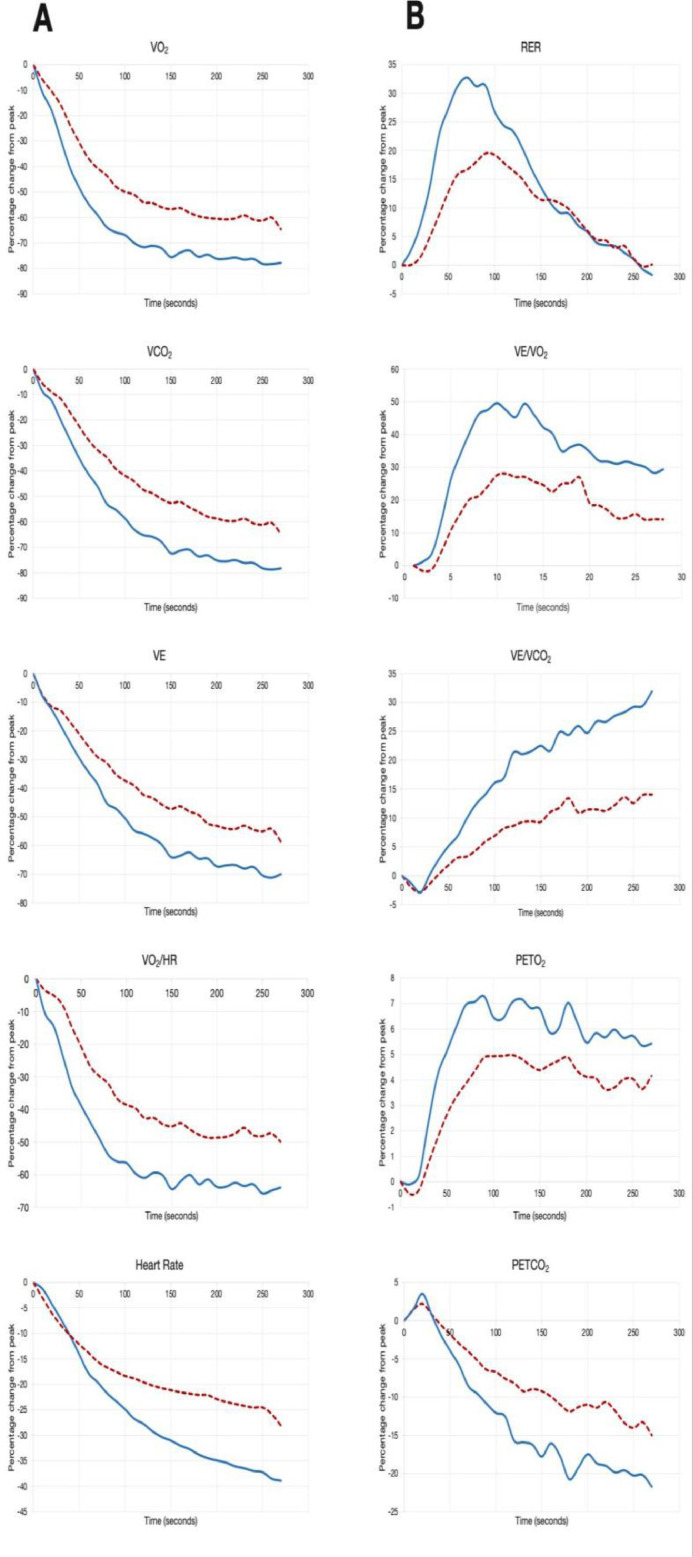
Panel A shows the average of VO_2_, VCO_2_, VE, VO_2_/HR and heart rate during recovery while panel B shows the average values of RQ, VE/VO_2_, VE/VCO_2_, PETO_2_ and PETCO_2_, data are expressed as percentage change from the value at peak exercise. Patients with a dotted red line, while healthy subjects are represented with a continuous blue line.

**Figure 2:**
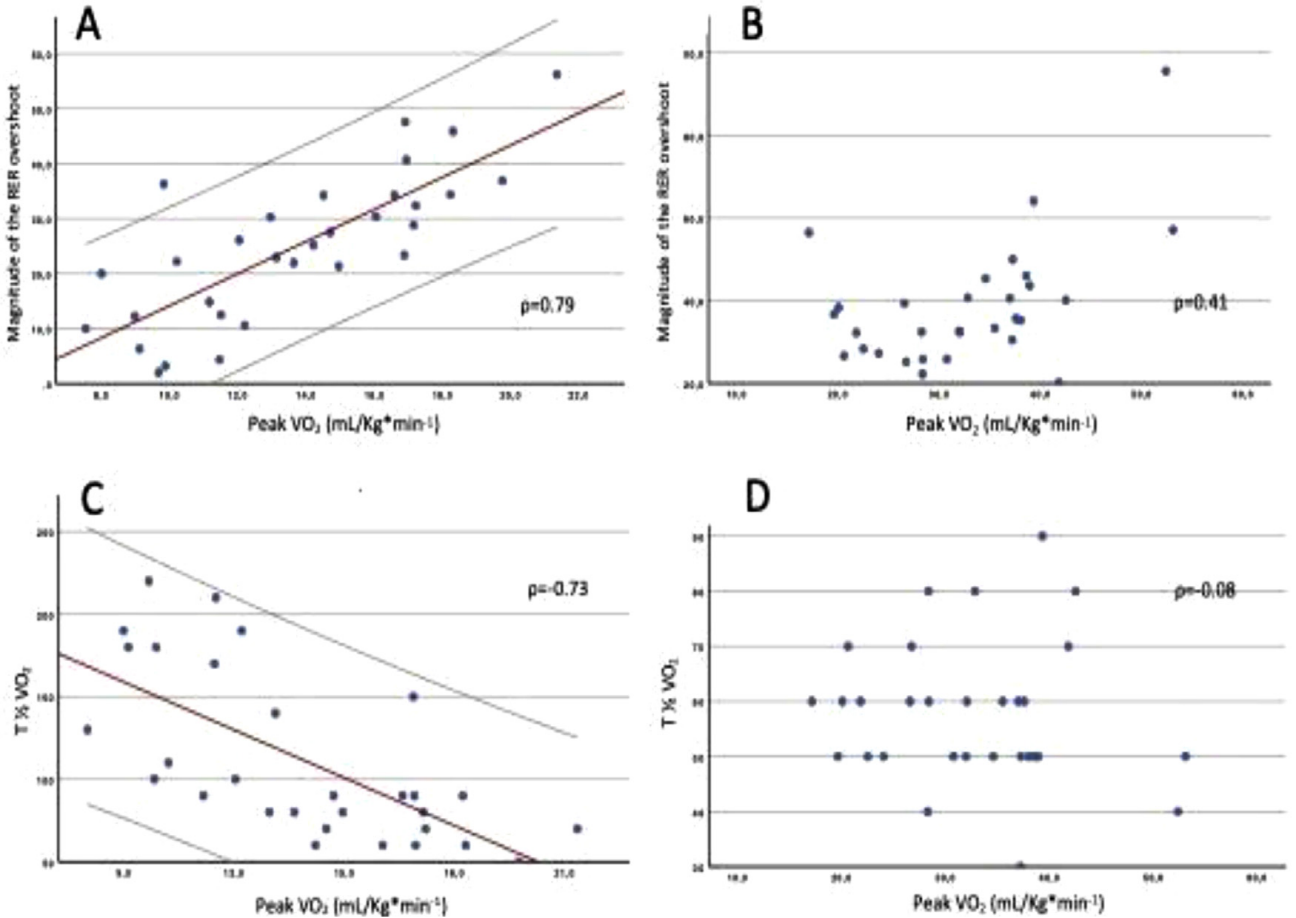
Correlations between T1/2 of VO_2_, magnitude of the RER overshoot and peak VO_2_ among with heart failure (panels A and C) and healthy subjects (panels C and D).

T1/2 for both VO_2_, VCO_2_ and VE were significantly longer in patients with CHF than in controls; similarly, the magnitude of the overshoot of VE/VO_2_, RER and PETO_2_ was lower in CHF. One patient (peak VO_2_ 7.8 mL/Kg/min^−1^) did not show an overshoot nor a recovery T½, one (peak VO_2_ 7.5 mL/Kg/min^−1^) did not show a recovery T½, and one (peak VO_2_ 9.7 mL/Kg/min^−1^) did not display an overshoot of PETO_2_. Three other patients did not show a recovery T½ of VE (peak VO_2_ of 11.5, 16.9 and 19.8 mL/Kg/min^−1^, respectively). Table 2 shows the recovery metrics in the two groups, while Figure 1, Figure 2 show the behavior of the CPX metrics during recovery.

**Table 2:** Recovery metrics for patients with congestive heart failure and healthy subjects.

	Patients	Controls	p
**Average recovery (s)**	248.8 ± 39.9	264.0 ± 29.3	<0.01
**T1/2 VO_2_ (s)**	111.3 ± 51.0	58.0 ± 13.2	<0.001
**T1/2 VCO_2_ (s)**	132.0 ± 38.8	74.3 ± 21.1	<0.001
**T1/2 VE (s)**	155.6 ± 45.5	96.7 ± 36.8	<0.001
**Overshoot of PETO_2_ (%)**	7.2 ± 3.3	10.1 ± 4.6	0.007
**Overshoot of VE/VO_2_ (%)**	41.9 ± 29.1	62.1 ± 17.7	0.002
**Overshoot of RER (%)**	25.0 ± 13.6	38.7 ± 15.1	<0.001

T1/2 VO_2_, Time to recovery of 50% of peak oxygen uptake; T1/2 VCO_2_, Time to recovery of 50% of peak carbon dioxide production; T1/2 VE, time to recovery of 50% of peak ventilation, PETO_2_, End-tidal oxygen tension; VE/VO_2_, Ventilatory equivalent for oxygen; RER, Respiratory Exchange Ratio (VCO_2_/VO_2_).

Recovery metrics showed statistically significant correlations with peak VO_2_. EF was not significantly correlated with the other variables in patients, while it was correlated with the PETO_2_ overshoot (*r* = 0.45, p = 0.01) in controls. The main correlations between the recovery metrics and CPX parameters are represented in Table 3.

**Table 3:** Correlations between the recovery parameters and the main CPX variables in patients with CHF.

	T_1/2_ VO_2_	T_1/2_ VCO_2_	T_1/2_ VE	PETO_2_ Mag	VE/VO_2_ Mag	RER Mag
**Age**	‒	‒	‒	‒	‒	‒
**BMI**	‒	‒	‒	‒	‒	‒
**Peak VO_2_ (mL/min)**	‒	‒	‒	‒	0.447^a^	0.580^b^
**Peak VO_2_ (mL/Kg/min)**	-0.732^b^	-0.604^b^	-0.473^a^	0.531^b^	0.604^b^	0.789^b^
**Peak VCO_2_ (mL/min)**	‒	‒	‒	‒	0.415^a^	0.526^b^
**Peak RER**	‒	‒	‒	‒	‒	‒
**Peak VE**	‒	‒	‒	‒	‒	0.495^b^
**VE/VCO_2_ Slope**	‒	‒	‒	-0.472^b^	‒	‒
**LVEF**	‒	‒	‒	‒	‒	‒
**VO_2_/HR**	-0.406^b^	-0.451^b^	-0.425^b^	‒	0.312^a^	0.617

BMI, Body Mass Index; LVEF, Left Ventricular Ejection Fraction; T1/2 VO_2_, Time to recovery of 50% of peak oxygen uptake; T1/2 VCO_2_, Time to recovery of 50% of peak carbon dioxide production; T1/2 VE, Time to recovery of 50% of peak ventilation; PETO_2_, End-tidal oxygen tension; VE/VO_2_, Ventilatory equivalent for oxygen; RER, Respiratory Exchange Ratio (VCO_2_/VO_2_).

a Correlation is significant at the 0.05 level.

b Correlation is significant at the 0.01 level.

The magnitude of the RER overshoot and the T½ of VO_2_ were the metrics more strongly correlated with peak VO_2_ (Pearson's *r* = 0.79 and Spearman's rho = -0.73 respectively, p = 0.01). Conversely, as shown in Figure 2, the correlation between these metrics and peak VO_2_ was weak in healthy subjects.

## Discussion

Despite the growing use of CPX in clinical practice, there is limited data on the value of assessing respiratory gas kinetics during the recovery phase after exercise. This is the first controlled study to provide a comprehensive assessment of relevant CPX metrics during recovery from peak exercise in a cohort of patients with CHF. The recovery of respiratory gases was found to be slower in patients with CHF than in healthy subjects, and this was correlated with a lower peak VO_2_ in CHF compared to controls.

Cohen-Solal et al. described the recovery of VO_2_ in patients with CHF as T½, showing that it was correlated with peak VO_2_, being reproducible and largely unaffected by exercise intensity (for >50% of the individual peak workload).^9^ Moreover, T½ VO_2_, VCO_2_ and VE increased with the worsening of CHF. The T½ of ratio of inorganic phosphate to creatine phosphate has been observed to be correlated with T½ VO_2_, reflecting an inability to replace peripheral energy stores.^9^ This inability to adequately respond to O_2_ deficits has been associated with reduced VO_2_/Work Rate slopes in patients compared to healthy subjects, suggesting lower aerobic efficiency.^13^ Similarly, Nanas et al. found the prolonged recovery of VO_2_ in patients with CHF correlated strongly with established indices of exercise capacity.^10^ A slower recovery of VO_2_ and cardiac output in patients with CHF has also been described by others.^11^^,^^22^^,^^23^

The main rationale underlying the potential usefulness of analyzing the VO_2_ kinetics during recovery is the hypothesis that the repayment of the oxygen debt is prolonged in patients with CHF.^13^ Furthermore, the delayed recovery of energy stores in peripheral muscles and the kinetics of recovery of oxygen consumption after exercise could be specific markers of the circulatory response during exercise, regardless of intensity.^9^ Even though gas exchange kinetics during recovery is likely to be the result of a complex balance of peripheral and central determinants of exercise performance, the mechanism underlying exercise intolerance typical of patients with CHF seems to be closely related to respiratory gas kinetics, observable in both during and in recovery from peak exercise. Furthermore, the recovery kinetics of VO_2_ seem to be correlated with indices of CRF but not with resting Left Ventricular Ejection Fraction (LVEF).^9^^,^^14^^,^^17^^,^^19^

Although the response of VO_2_ on-kinetics during exercise has been proposed as an important marker for CPX evaluation in different chronic diseases,^24^, ^25^, ^26^ it has been demonstrated in patients with CHF that during submaximal exercise the recovery kinetics of VO_2_ is more reproducible than the VO_2_ on-kinetics.^27^ The recovery response can help discriminate patients with CHF from their healthy counterparts, even in presence of similar VO_2_ on-kinetics.^27^

More recently, the knowledge around this topic has been implemented describing the pattern of other CPX metrics (RER, PETO_2_ and VE/VO_2_) during recovery, which commonly displays an “overshoot” and seems to be reduced in magnitude among patients with CHF. A good correlation was found between the magnitude of the overshoot and common indices of CRF. However, no correlation was found with LVEF at rest, suggesting at best a weak relationship with resting cardiac function.^19^

In the present study, the behavior of recovery variables that display an “overshoot” (RER, VE/VO_2_ and PETO_2_) supports the observations of previous studies. In particular, Takayanagi et al. found the presence of a recovery overshoot of the aforementioned metrics in 100% of their cardiac patients.^19^ In the present study, two patients did not display an overshoot of PETO_2_ while one patient did not display an overshoot of RER or VE/VO_2_. These subjects were among those with the most severe disease.

These data demonstrate that the assessment of recovery kinetics of respiratory gases is feasible in a clinical setting with results that are reasonably comparable to those obtained during experimental studies. Moreover, the present results compare common indices of recovery (such as T1/2) with novel markers (e.g., the magnitude of the overshoot), demonstrating that the magnitude of the overshoot of the RER is the variable with the strongest correlation with peak VO_2_. This suggests the potential utility of CPX recovery alongside the standard clinical evaluation of these patients. The fact that two patients among those in Weber's class D didn't show an overshoot of PETO_2_, RER, and VE/VO_2_ is a novel finding of the present study. It is possible that the absence of an overshoot could be a marker for more severe impairment of the cardiorespiratory response during exercise.

Among patients with CHF, the recovery variables assessed in the present study showed generally a good correlation with peak VO_2_, particularly T½ VO_2_ and the overshoot of RER. Conversely, there was a weak correlation between these metrics in controls.

The current data support the hypothesis that recovery metrics are not sole derivatives of peak VO_2_ but rather reflect other physiologic mechanisms (such as the capacity to resynthesize PCr). This might be associated with a deficit in the replenishing of oxygen accumulated during exercise due to impaired cardiac output or local circulatory dysfunction.^9^^,^^13^^,^^28^ Thus, it seems reasonable to hypothesize that the “anaerobic burden” of exercise incurred by patients with CHF carries detrimental consequences during and after exercise, resulting in lower VO_2_/WR slopes, exertional dyspnea, and resulting in slower recovery kinetics. These considerations are further supported by the lack of strong correlations between resting ventricular function and the recovery parameters in both patients and controls. Studies that investigate muscle metabolism during and after exercise in these patients would help to answer some of these questions.^19^

Compared to the correlation between the recovery variables and peak VO_2_, the correlation between the recovery metrics and VE/VCO_2_ appeared to be weaker. This finding was unexpected since previous studies showed significant correlations of recovery metrics with VE/VCO_2_ slope.^19^ However, it is possible that different methods have been used to assess VE/VCO_2_, which could result in large differences between the studies.

Although assessing the prognostic significance of these recovery metrics was not the purpose of this study, their prognostic utility may be inferred based on previous literature. In fact, De Groote et al. found that in patients with dilated cardiomyopathy and moderate exercise intolerance, the ratio between total oxygen consumption during exercise and during recovery was an independent predictor of survival together with left ventricular ejection fraction.^14^ Other authors investigated T½ VO_2_ in patients with CHF, showing that it was significantly associated with prognosis.^16^^,^^17^ More recently, Fortin et al. found VO_2_ recovery as a stronger predictor of death, heart transplantation, and mechanical heart implantation than well-established prognostic markers such as peak VO_2_ and Heart Failure Survival Score (HFSS).^15^ Finally, Bailey et al. analyzed the time from the end of loaded exercise until VO_2_ permanently fell below peak VO_2_, demonstrating an association with worse transplant-free survival.^13^ Collectively, these results suggest that integrating the assessment of recovery during routine CPX has promise for increasing the diagnostic and prognostic precision of functional evaluation in patients with CHF. To be implemented into clinical practice, future studies on the underlying pathophysiological mechanisms of recovery in healthy subjects and patients will be needed, together with standardization of recovery metrics.

### Future perspectives

Cardiac rehabilitation programs are currently recommended by major scientific societies and guidelines for the management of CHF, with different modalities and types of exercise training.^29^ An important question regarding the recovery phase will be whether and to what extent cardiopulmonary adaptations that occur after exercise could depend on the training program. In a context where different modalities of exercise are used for cardiac rehabilitation, including short bouts of exercise interspersed with relatively short recovery phases such as interval training, it can be hypothesized that adding recovery data to the functional evaluation of patients with CHF could help in tailoring an exercise prescription. Indeed, the recovery phase during interval training could be adapted based on the CPX recovery responses in order to optimize muscular recovery and minimize exertional dyspnea.

### Limitations

This was a retrospective study based on CPX evaluations performed for clinical purposes compared to health controls. Although the main objective of the study was to comprehensively describe CPX parameters during recovery in a population of patients with CHF, definitive conclusions on the prognostic significance of these recovery markers cannot be drawn from the present data. Future integration of the parameters studied with prognostic data will permit a better understanding of the impact of impaired recovery on disease progression as well as on survival and/or hospitalizations.

Moreover, since the pathophysiology behind the recovery delay is thought to be closely connected to the O_2_ deficit and thus to the “anaerobic burden” during exercise, the lack of data on lactic acid accumulation limits the understanding of how and to what extent the recovery parameters reflect the relative aerobic and anaerobic contributions in patients with CHF.

Finally, although most participants fall into the ‘overweight’ category based on BMI, the CHF group had a significantly higher average BMI than the control group (27.9±5.0 compared to 24.5±2.9 Kg/m^2^, p=0.002). This difference could affect exercise tolerance in cycle ergometry and have some influence on the interpretation of the results and conclusions of the current trial.

## Conclusions

The present study strengthens the evidence on the blunted recovery of VO_2_ in patients with CHF and for the first time provides data on the recovery of respiratory gas kinetics (VO_2_, VCO_2_) and VE together with data on the recovery of those parameters that tend to show an overshoot after exercise (PETO_2_, RER, VE/VO_2_), showing good reproducibility of this kind of analysis in a clinical setting. The results strengthen previous evidence that reported a slower recovery of VO_2_, VCO_2_ and VE after exercise in patients with CHF, and a reduced magnitude of the recovery overshoot of RER, PETO_2_ and VE/VO_2_. A good correlation between recovery parameters and peak VO_2_ was observed in patients with CHF but not in controls, suggesting a greater role of aerobic capacity in determining the recovery kinetics of subjects belonging to lower classes of cardiorespiratory fitness. A more standardized evaluation of this phase could provide important additional information on patient's functional capacity and help tailor their exercise prescription for cardiac rehabilitation programs.

## Declaration of Competing Interest

The authors declare no conflicts of interest.
